# Investigation of the Relationship Between Psychiatry Visit and Suicide After Deliberate Self-harm: Longitudinal National Cohort Study

**DOI:** 10.2196/41261

**Published:** 2023-04-12

**Authors:** Hye Hyeon Kim, Chanyoung Ko, Ji Ae Park, In Han Song, Yu Rang Park

**Affiliations:** 1 Department of Biomedical Systems Informatics, Yonsei University College of Medicine Seoul Republic of Korea; 2 Health & Mental Health Lab, Yonsei University Graduate School of Social Welfare Seoul Republic of Korea; 3 Institute for Convergence Science Academy, Yonsei University Seoul Republic of Korea

**Keywords:** deliberate self-harm, suicide, psychiatry, suicidal, death, mortality, psychiatric, Korea

## Abstract

**Background:**

Deliberate self-harm (DSH) along with old age, physical disability, and low socioeconomic status are well-known contributors to suicide-related deaths. In recent years, South Korea has the highest suicide death rate among all Organization for Economic Co-operation and Development countries. Owing to the difficulty of accessing data of individuals with DSH behavior who died by suicide, the factors associated with suicide death in these high-risk individuals have not been sufficiently explored. There have been conflicting findings with regard to the relationship between previous psychiatric visits and suicidal death.

**Objective:**

We aimed to address the following 3 questions: Are there considerable differences in demographics, socioeconomic status, and clinical features in individuals who received psychiatric diagnosis (either before DSH or after DSH event) and those who did not? Does receiving a psychiatric diagnosis from the Department of Psychiatry, as opposed to other departments, affect survival? and Which factors related to DSH contribute to deaths by suicide?

**Methods:**

We used the Korean National Health Insurance Service Database to design a cohort of 5640 individuals (3067/5640, 54.38% women) who visited the hospital for DSH (International Classification of Diseases codes X60-X84) between 2002 and 2020. We analyzed whether there were significant differences among subgroups of individuals with DSH behavior based on psychiatric diagnosis status (whether they had received a psychiatric diagnosis, either before or after the DSH event) and the department from which they had received the psychiatric diagnosis. Another main outcome of the study was death by suicide. Cox regression models yielded hazard ratios (HRs) for suicide risk. Patterns were plotted using Kaplan-Meier survival curves.

**Results:**

There were significant differences in all factors including demographic, health-related, socioeconomic, and survival variables among the groups that were classified according to psychiatric diagnosis status (*P*<.001). The group that did not receive a psychiatric diagnosis had the lowest survival rate (867/1064, 81.48%). Analysis drawn using different departments from where the individual had received a psychiatric diagnosis showed statistically significant differences in all features of interest (*P*<.001). The group that had received psychiatric diagnoses from the Department of Psychiatry had the highest survival rate (888/951, 93.4%). These findings were confirmed using the Kaplan-Meier survival curves (*P*<.001). The severity of DSH (HR 4.31, 95% CI 3.55-5.26) was the most significant contributor to suicide death, followed by psychiatric diagnosis status (HR 1.84, 95% CI 1.47-2.30).

**Conclusions:**

Receiving psychiatric assessment from a health care professional, especially a psychiatrist, reduces suicide death in individuals who had deliberately harmed themselves before. The key characteristics of individuals with DSH behavior who die by suicide are male sex, middle age, comorbid physical disabilities, and higher socioeconomic status.

## Introduction

Deliberate self-harm (DSH) is a behavior in which one intentionally harms oneself either by means of nonfatal methods (wrist cutting or self-poisoning via medication with minimal adverse side effects) or fatal methods (the use of firearms or pesticides or hanging) [1,2]. It is a global health problem and is clinically important because individuals who commit DSH are at an increased risk of death by suicide [3-8]. A history of DSH is not only a marker of short-term suicide and subsequent self-harm but is also associated with poor treatment prognosis and noncompliance [2].

Every suicide case can be viewed as a DSH case, but not all DSH cases necessarily lead to suicide [2]. Therefore, it is difficult to measure DSH rates from nationwide statistical polls, which only report the number of deaths by suicide [9]. Previous studies on DSH used cohorts of small to moderate sizes based on surveys geared toward a particular population or extracted from national medical service use data using the International Classification of Diseases, 10th Revision (ICD-10) codes for DSH diagnoses and the cause of death information derived from linkage to the National Death Index [1,10,11]. Furthermore, acquiring a nationwide self-harm cohort assembly is difficult because not every individual with DSH behavior uses medical services or receives clinical diagnoses for DSH [12,13]. Although studying these individuals at the national level would be technically improbable, attempting to do so using patient information collected from every hospital in the respective country may still reveal valuable information. This information could be generalized to better understand these high-risk individuals, allowing for more suicide prevention opportunities.

Previous literature informs us of the factors contributing to deaths by suicide. These include sociodemographic factors such as poor income class; old age; male sex; and clinical data such as having received a psychiatric diagnosis, previous suicide attempts, substance abuse history, and having physical disabilities [3,5,6,12,14-19]. Among various physical disabilities and medical conditions, disease states affecting activities of daily living and overall function such as malignancies, musculoskeletal disorders, pulmonary conditions (ie, chronic obstructive pulmonary disease and asthma), and neurological conditions (ie, stroke) were not only associated with severe mental illnesses and increased health service usage [20] but also act as important risk factors for suicide in both men and women [16,21]. According to one study, older adults with physical disabilities who die by suicide often consult their physicians within weeks of their death [12]. Physical symptoms such as pain are often the focus of these visits, and mental distress and suicidal and self-harming ideations are often missed [15].

Although there is much evidence for this important connection between physical disabilities and deaths by suicide, nonpsychiatric health care professionals tend to take ambivalent stances on how to address mental health issues with their patients, as suggested by a few previous studies [22-24]. This ambivalence most likely stems from anticipated difficulties in dealing with patients with mental health conditions [25], and the scarcity of protocols for the assessment and management of mental health crises in nonpsychiatric patients is thought to be fueling the discomfort of actively screening for mental health issues and suicide risk in this population. Currently, questionnaires and scales for screening depression and anxiety, such as the Patient Health Questionnaire-9 [26], and suicidal ideation, such as the Columbia-Suicide Severity Rating Scale [27], and protocols that not only assess but also offer suicide-specific interventions, such as the Collaborative Assessment and Management of Suicidality [28], are clinical tools exclusively used in psychiatric emergency settings, such as a psychiatric ward or emergency room [29]. Unless receiving extensive training for how to administer these tools, it is very unlikely that nonpsychiatric health care professionals would be able to successfully make use of them, which was a point addressed in one study that investigated the possibility of implementing mental health risk assessment tools using digital resources [30]. In all parts of the world, there appears to be a lack of evidence-based, comprehensive guides for nonpsychiatric health care professionals in mental health and suicide risk assessment in nonpsychiatric patients; therefore, this area requires much more attention and investigation. By studying individuals with DSH behavior who failed to receive psychiatric assessment, whom we expect to also have physical disabilities, we may be able to find important clues for developing a suicide risk assessment in a nonpsychiatric patient protocol geared toward nonpsychiatric health care professionals.

Suicide is a greater issue for South Korea, which has been rated as the leading country for suicide among all Organization for Economic Co-operation and Development countries for several years [31]. To date, 3 studies have used Korean National Death Registration data or National Health Insurance (NHI) service data, from 2002 to 2013, to study the factors associated with suicide rate trends in South Korea [8,18,32]. Choi et al [8] and Chen et al [18], using 2 different data sets that were both still representative of the entire South Korean population, reported that old age, low socioeconomic status, and visiting medical facilities with ICD-10 F00-F99 codes (psychiatric diagnoses and mental and behavioral disorders) were associated with an increased risk of suicide completion. A study conducted by Kim et al [32] investigated the prevalence of depression in South Korea and provided evidence that an increased prevalence of depression contributed to an increased risk of suicide. Previous literature on suicide-related deaths in South Korea neither investigated nor used nationwide all-hospital patient data on self-harm. Moreover, the authors did not use a strict operational definition of death by suicide to study individuals with DSH behavior in South Korea. Therefore, a more thorough investigation of individuals with DSH behavior and the factors contributing to the increased rate of suicide is warranted.

**Objectives**

This study aimed to address the following questions: (1) Are there significant differences in demographics, socioeconomic status, and clinical features in individuals who received a psychiatric diagnosis (either before DSH or after the DSH event) and those who did not? (2) Does receiving a psychiatric diagnosis from the Department of Psychiatry as opposed to other departments affect survival? and (3) Which factors related to DSH contribute to death by suicide?

## Methods

### Data Sources

We used the Korean National Health Insurance Service (KNHIS) database [33], an administrative database based on health insurance claims from the entire South Korean population, to analyze the characteristics of patients with DSH and suicidal behavior. In this retrospective study, we constructed a DSH cohort to identify patients who visited the hospital for more than one DSH episode from January 2002 to December 2020. DSH was defined using ICD-10 codes for intentional self-harm (X60-X84).

The KNHIS is a universal coverage health insurance system, which includes detailed treatment practices and prescriptions based on the fee-for-service payment model as well as the health information of all citizens who have signed up for national medical insurance in South Korea [34]. Almost all South Koreans (97%) are enrolled in the KNHIS and most receive medical treatment at least once a year [33]. Data included public data on health care use such as disease diagnoses; drug prescriptions and procedures; national health examination results, including smoking, drinking, physical measurements, and body measurements; and demographic and socioeconomic variables such as age, sex, income rank, household location, disability, and mortality of the entire Korean population. In particular, within the health insurance claim data, diagnoses were coded in compliance with the ICD-10 [34,35]. However, because DSH is a very sensitive subject, some data related to personal information, such as personal data of clinics and household location, detailed drug prescriptions, and treatments, were not available. Statistics Korea links and provides the cause of death statistics [36]. We asked Statistics Korea to link the cause and date of death of all patients with DSH. The KNHIS data were linked to the cause of death statistics based on the resident number for patients.

### Study Design

This study was designed to identify and analyze how differences in suicide survival and demographic, socioeconomic, and health-related variables vary depending on psychiatric diagnosis for patients who were admitted to the hospital after DSH. We chose certain factors, previously shown to have moderate to high correlation with DSH or death by suicide, as target variables for analysis using the KNHIS database [2,4,11,14,37]. Through this process, a total of 11 variables from the KNHIS database were extracted for in-depth analyses: age, sex, drinking, smoking, cancer, physical disability, insurance type, psychiatric disorders or psychiatric diagnosis, self-harm treatment setting, self-harm method, and somatic disorders or Charlson comorbidity index (CCI; Multimedia Appendix 1). Before performing the main analyses, we preprocessed the data using the following 4 steps.

First, we included suicide survival status and DSH severity in the DSH-related factors. Death status was classified by confirming the date of death, and suicide death was classified by confirming the diagnostic code with X60-X84 from the ICD-10. We also classified the severity of DSH using the DSH method. DSH attempts with drugs (X60-X64) and cutting (X78) were defined as nonfatal DSH. In addition, we defined DSH attempts as fatal DSH including hanging (X70), pesticides (X68), or jumping from heights (X80), according to previous studies [38,39].

Second, we included age and sex in the demographic factors. We excluded patients under the age of 10 years because the cause of death could be accidental, and death by suicide was perceived as intentional after the age of 10 years [40]. We analyzed the differences between the groups by grouping the ages by 10 years.

Third, we included CCI, cancer, psychiatric visits, and disability in the health-related factors [36]. CCI is a widely used comorbidity correction method that involves assigning a specific weight of 1 to 6 points to 19 diseases identified by medical record surveys and then adjusting the sum of these weights [36]. This was used to confirm the risk of DSH according to the severity of the disease. We also categorized the patients into 2 groups: those who had at least one cancer diagnosis and those who had none, regardless of the type of cancer. For psychiatric visits, we included all events when patients had been given a psychiatric diagnosis at least once, not only in the psychiatric department but also in other departments. Disability was classified into 6 grades and was used as a substitute indicator of mobility. These grades were also classified into 3 categories: severe (grades 1-3), mild (grades 4-6), and no disability [41].

Finally, we included smoking, drinking, and NHI as the socioeconomic factors. For smoking and drinking, although the units of measurement were different according to the type of alcohol and tobacco, we categorized patients into 3 groups according to its severity: (1) *severe*, when substantial use level required health care provider or expert help, (2) *moderate*, when substantial use level was less severe but abstinence was recommended, and (3) *NA*, when a related item was not measured because of nonsubstantial users or unknown reasons. For NHI, we classified people into 2 groups: Medical Aid beneficiaries (Medical benefit) who are covered by the Medical Aid program as a low-income population and NHI members with employee health insurance and local-subscriber health insurance (employee or local) above the threshold of 50% of the median income who are covered by the NHI program [42].

**Figure 1:**
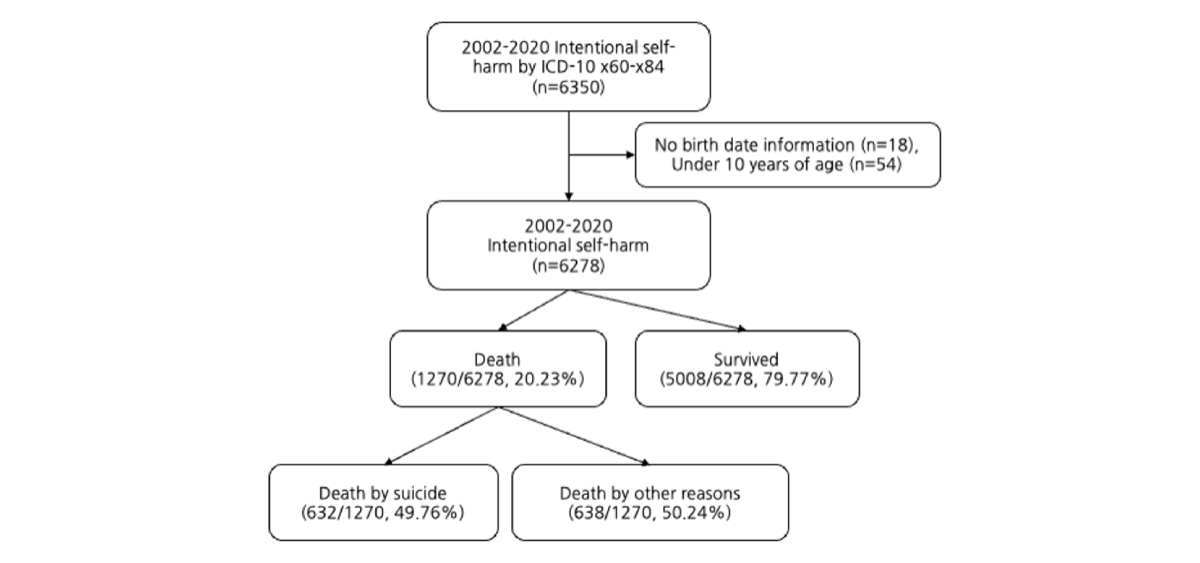
Patient selection flow. ICD-10: International Classification of Diseases, 10th Revision.

Of the 6350 patients who committed DSH, 18 (0.28%) patients with missing birth dates and 54 (0.85%) patients aged <10 years were excluded. The cohort database in this study comprised 6278 patients who had experienced more than one deliberate episode from January 2002 to December 2020, and among the cohort participants, 5008 survivors and 632 suicide deaths were selected for the analysis (Figure 1).

To determine how psychiatry and its treatment affect DSH, we divided the patients into the following groups: (1) those who self-harmed before receiving a psychiatric diagnosis, (2) those who self-harmed after receiving a psychiatric diagnosis, and (3) those who did not receive a psychiatric diagnosis. We then analyzed whether these groups showed significant differences in their features.

A psychiatric diagnosis can be received in other departments through practices such as the prescription of psychiatric drugs. We also analyzed in detail whether there were significant differences between the groups classified according to the department providing the psychiatric diagnosis. All patients were classified into five groups as follows: (1) psychiatric diagnosis received only from psychiatry, (2) psychiatric diagnosis received from other departments and then from psychiatry, (3) psychiatric diagnosis received from psychiatry and then from other departments, (4) psychiatric diagnosis received from other departments only, and (5) no psychiatric diagnosis. Next, we analyzed whether there were significant differences between the groups.

### Statistical Analysis

The mean and SD or the median and range were used to express continuous variables. ANOVA was used to compare categorical independent variables (with 2 or more categories) and a normally distributed interval-dependent variable. The Kruskal-Wallis test was used when 1 independent variable had 2 or more levels and an ordinal dependent variable. The cumulative incidence of events was calculated using the Kaplan-Meier estimator of the relevant survival function and graphed with 95% CIs. Hazard ratios (HRs) were calculated using a Cox proportional hazards model. To identify the risk factors possibly affecting suicide after DSH, we first conducted a univariate analysis using a logistic regression model procedure for 11 variables. In the multivariate linear regression analysis, only variables that were statistically significant (*P*<.05) through univariate regression analysis were selected, and their effect on suicidal risk was evaluated. Variables found to be significant in the univariate analysis were included in the multivariate analysis using the Cox proportional hazards model [43]. Among the Cox regression analysis results, the HRs and 95% CIs were reported. The threshold for statistical significance was set at *P*<.05. The R software was used to conduct all statistical analyses (version 3.6.3; R Foundation for Statistical Computing).

### Ethics Approval

This retrospective study was approved by the institutional review board of Severance Hospital, Yonsei University College of Medicine, and the requirement for informed consent was waived (institutional review board number 4-2021-0950). As this was a retrospective study, the need for informed consent was waived because it used collected data that were anonymously managed at all stages, including data cleansing and statistical analyses.

## Results

### Patient Characteristics

A total of 5640 patients were included in this study, of which 2573 (45.62%) were male, and the remaining were female. Of the 5640 patients, 4576 (81.13%) patients received a psychiatric diagnosis between January 2002 and December 2020, with 3821 (67.75%) patients receiving a psychiatric diagnosis before self-harm. In total, 1064 (18.87%) patients did not receive any psychiatric diagnosis or treatment.

There were significant differences in all features, including demographic, health-related, socioeconomic, and survival variables between the groups (*P*<.001; Table 1). Most notably, there was a significant difference in survival rates sequentially in the group that did not receive a psychiatric diagnosis (81.48%), followed by one group that received psychiatric diagnosis and later self-harmed (89.06%), and one group that self-harmed before receiving the psychiatric diagnosis (97.7%). As each group had distinct characteristics, we described the characteristics of each group in the order of the suicide survival rate as follows.

**Table 1:** Characteristics of individuals with deliberate self-harm (DSH) behavior based on psychiatric diagnosis status (receiving at least one psychiatric diagnosis either before or after DSH event or not receiving any at all).

					Self-harm before receiving psychiatric diagnosis (n=755, 13.39%)		Self-harm after receiving psychiatric diagnosis (n=3821, 67.75%)		No psychiatric diagnosis (n=1064, 18.87%)		Total (N=5640)		*P* value		
**Suicide survival status, n (%)**															<.001
			Survived		738 (97.7)		3403 (89.06)		867 (81.48)		5008 (88.79)				
			Death by suicide		17 (2.3)		418 (10.94)		197 (18.52)		632 (11.21)				
**Self-harm severity, n (%)**															<.001
			Fatal		364 (48.2)		1393 (36.46)		600 (56.39)		2357 (41.79)				
			Nonfatal		391 (51.8)		2428 (63.54)		464 (43.61)		3283 (58.21)				
**Demographic factors**															
	**Sex, n (%)**													<.001	
		Male		399 (52.8)		1505 (39.39)		669 (62.88)		2573 (45.62)					
		Female		356 (47.2)		2316 (60.61)		395 (37.12)		3067 (54.38)					
	Age, median (range)			40.0 (25.0-53.0)		48.0 (32.0-63.0)		43.0 (29.0-54.0)		46.0 (30.0-59.0)		<.001			
	**Age group, n (%)**													<.001	
		10-19		104 (13.8)		264 (6.91)		123 (11.56)		491 (8.71)					
		20-29		139 (18.4)		562 (14.71)		154 (14.47)		855 (15.16)					
		30-39		134 (17.7)		523 (13.69)		192 (18.05)		849 (15.05)					
		40-49		133 (17.6)		670 (17.53)		213 (20.02)		1016 (18.01)					
		50-59		132 (17.5)		686 (17.95)		200 (18.80)		1018 (18.05)					
		60-69		61 (8.1)		430 (11.25)		88 (8.27)		579 (10.27)					
		70-79		31 (4.1)		405 (10.60)		73 (6.86)		509 (9.02)					
		>80		21 (2.8)		281 (7.35)		21 (1.97)		323 (5.73)					
**Health-related factor**															
	Charlson comorbidity index score, median (range)			0.0 (0.0-2.0)		1.0 (0.0-3.0)		1.0 (0.0-2.0)		1.0 (0.0-3.0)		<.001			
	Cancer, n (%)			298 (39.5)		1827 (47.81)		363 (34.12)		2488 (44.11)		<.001			
	Psychiatric visit, n (%)			540 (71.5)		3261 (85.34)		0 (0.00)		3801 (67.39)		<.001			
	**Disability, n (%)**													<.001	
		Mild		43 (5.7)		353 (9.24)		52 (4.89)		448 (7.94)					
		Severe		32 (4.2)		255 (6.67)		21 (1.97)		308 (5.46)					
		No		680 (90.1)		3213 (84.09)		991 (93.14)		4884 (86.60)					
**Socioeconomic factor, n (%)**															
	**Smoking**													<.001	
		Moderate		283 (37.5)		1731 (45.30)		403 (37.88)		2417 (42.85)					
		Heavy		171 (22.6)		702 (18.37)		252 (23.68)		1125 (19.95)					
		N/A^a^		301 (39.9)		1388 (36.33)		409 (38.44)		2098 (37.20)					
	**Drinking**													<.001	
		Moderate		265 (35.1)		1697 (44.41)		376 (35.34)		2338 (41.45)					
		Heavy		190 (25.2)		730 (19.11)		278 (26.13)		1198 (21.24)					
		N/A		300 (39.7)		1394 (36.48)		410 (38.53)		2104 (37.31)					
	**National Health Insurance**													<.001	
		Employee or local		718 (95.1)		3343 (87.49)		1029 (96.71)		5090 (90.25)					
		Medical benefit		37 (4.9)		478 (12.51)		35 (3.29)		550 (9.75)					

^a^N/A: not applicable.

First, the group without a psychiatric diagnosis had the lowest suicide survival rate (867/1064, 81.48%) and highest rate of fatal self-harm (600/1064, 56.39%). A total of 1064 patients were included in the group, 197 (18.52%) of whom died by suicide. The median age was 43.0 (range 29.0-54.0) years, and 669 (62.88%) patients were male. This group was mainly composed of middle-age men (605/1064, 66.87%) in their 30s and 50s. Regarding health-related factors, none of them had a history of psychiatric visits, and many (991/1064, 93.14%) had no disability. Regarding socioeconomic factors, most had employee or local insurance (1029/1064, 96.71%) and had the highest rate of heavy smokers and drinkers among the 3 groups.

Second, the group that self-harmed after receiving a psychiatric diagnosis had the highest rate of DSH using the nonfatal method (2428/3821, 63.54%). A total of 3821 patients were included in this group, 418 of whom died by suicide. Regarding demographic factors, there was a high proportion of older women (aged >60 years) and the median age was 48.0 (range 32.0-63.0) years. They had the highest rates of cancer (1827/3821, 47.81%) and psychiatric visit history (3261/3821, 85.34%) among the 3 groups. A history of disability was noted in 608 (608/3821, 15.91%) patients. Socioeconomic factors had the highest rates for medical aid benefit insurance (478/3821, 12.51%), moderate smoking (1731/3821, 45.30%), and drinking (1697/3821, 44.41%).

Finally, the group that self-harmed before receiving a psychiatric diagnosis, that is, the group that self-harmed before clinical visits, had the highest suicide survival rate (738/755, 97.7%), with a high rate of nonfatal self-harm (391/755, 51.8%). A total of 755 patients were included in this group, 17 of whom died by suicide. The median age of the patients was 40.0 (range 25.0-53.0) years, and 399 (52.8%) patients were male. They had a higher rate of people in their 10s and 20s (32.2%) than other age groups. Cancer, disability history, and psychiatric visit history was noted in 298 (39.5%), 37 (4.8%), 75 (9.8%), and 540 (71.5%) patients, respectively. Regarding socioeconomic factors, most participants had employee or local insurance (718/755, 95.1%), and there were more smokers (454/755, 60.1%) and drinkers (455/755, 60.3%).

### Characteristics of Individuals With DSH Behavior

We also classified all patients into 5 subgroups based on the medical department they had received a psychiatric diagnosis from (Department of Psychiatry vs other departments) to determine whether psychiatric evaluation from the Department of Psychiatry as opposed to a nonpsychiatric department affected the outcome (Table 2). All variables were significantly different among the 5 groups (*P*<.001). In particular, these groups significantly differed in suicide rates from the group treated at the psychiatric clinic only, with the highest suicide survival rate (888/951, 93.4%) in the group with no psychiatric diagnosis and the lowest suicide survival rate (867/1064, 81.48%) in the following order.

**Table 2:** Comparison of individuals with deliberate self-harm by the different departments through which one received a psychiatric diagnosis.

Variable				Psychiatric diagnosis from psychiatry (n=951, 16.86%)		Psychiatric diagnosis from other departments first, then psychiatry (n=1330, 23.58%)		Psychiatric diagnosis from psychiatry first, then other departments (n=1520, 26.95%)		Psychiatric diagnosis from other departments (n=775, 13.74%)		No psychiatric diagnosis (n=1064, 18.87%)		Total (N=5640)		*P* value	
**Survival status, n (%)**																	<.001
	Survived		888 (93.4)		1212 (91.13)		1366 (89.87)		675 (87.1)		867 (81.48)		5008 (88.79)				
	Death by suicide		63 (6.6)		118 (8.87)		154 (10.13)		100 (12.9)		197 (18.52)		632 (11.21)				
**Self-harm severity, n (%)**																	<.001
	Fatal		615 (64.7)		877 (65.94)		946 (62.24)		381 (49.2)		464 (43.61)		2357 (41.79)				
	Nonfatal		336 (35.3)		453 (34.06)		574 (37.76)		394 (50.8)		600 (56.39)		3283 (58.21)				
**Demographic factors**																	
	**Sex, n (%)**																<.001
		Male	432 (45.4)		504 (37.89)		562 (36.97)		406 (52.4)		669 (62.88)		2573 (45.62)				
		Female	519 (54.6)		826 (62.11)		958 (63.03)		369 (47.6)		395 (37.12)		3067 (54.38)				
	Age (years), median (range)		32.0 (21.0-47.0)		45.0 (30.0-59.0)		52.0 (38.0-68.0)		53.0 (40.0-66.0)		43.0 (29.0-54.0)		51.0 (40.0-64.0)		<.001		
	**Age groups, n (%)**																<.001
		10-19	178 (18.6)		100 (7.52)		64 (4.21)		26 (3.3)		123 (11.56)		491 (8.71)				
		20-29	247 (25.8)		227 (17.07)		163 (10.72)		64 (8.2)		154 (14.47)		855 (15.16)				
		30-39	170 (17.7)		200 (15.04)		191 (12.57)		96 (12.4)		192 (18.05)		849 (15.05)				
		40-49	153 (16.0)		241 (18.12)		266 (17.50)		143 (18.4)		213 20.02)		1016 (18.01)				
		50-59	101 (10.5)		230 (17.29)		311 (20.46)		176 (22.7)		200 (18.80)		1018 (18.05)				
		60-69	54 (5.6)		155 (11.65)		180 (11.84)		102 (13.1)		88 (8.27)		579 (10.27)				
		70-79	32 (3.3)		113 (8.50)		202 (13.29)		89 (11.5)		73 (6.86)		509 (9.02)				
		>80	16 (1.7)		64 (4.81)		143 (9.41)		79 (10.1)		21 (1.97)		323 (5.73)				
	**Medical history–related factor**																
		Cancer, n (%)	322 (33.9)		641 (48.20)		812 (53.42)		350 (45.2)		363 (34.12)		2488 (44.11)		<.001		
		Charlson comorbidity index score, median (range)	0.0 (0.0-1.0)		1.0 (0.0-2.0)		2.0 (0.0-3.0)		2.0 (0.0-3.0)		1.0 (0.0-2.0)		2.0 (0.0-3.0)		<.001		
	**Disability status, n (%)**																<.001
		Mild	24 (2.5)		106 (7.97)		173 (11.38)		83 (10.7)		52 (4.89)		438 (7.77)				
		Severe	32 (3.3)		103 (7.74)		118 (7.76)		35 (4.5)		21 (1.97)		309 (5.48)				
		No	895 (94.1)		1121 (84.29)		1229 (80.86)		657 (84.8)		991 (93.14)		4893 (86.76)				
**Socioeconomic factors, n (%)**																	
	**Smoking**																<.001
		Moderate	278 (29.2)		574 (43.16)		772 (50.79)		390 (50.3)		403 (37.88)		2417 (42.85)				
		Heavy	163 (17.1)		250 (18.80)		291 (19.14)		169 (21.8)		252 (23.68)		1125 (19.95)				
		N/A^a^	510 (53.6)		506 (38.04)		457 (30.07)		216 (27.9)		409 (38.44)		2098 (37.20)				
	**Drinking**																<.001
		Moderate	250 (26.3)		582 (43.76)		775 (50.99)		355 (45.8)		376 (35.34)		2338 (41.45)				
		Heavy	192 (20.2)		241 (18.12)		284 (18.68)		203 (26.2)		278 (26.13)		1198 (21.24)				
		N/A	509 (53.5)		507 (38.12)		461 (30.33)		217 (28.0)		410 (38.53)		2104 (37.30)				
	**National Health Insurance**																<.001
		Employee or local	928 (97.6)		1160 (87.22)		1273 (83.75)		700 (90.3)		1029 (96.71)		5090 (90.25)				
		Medical benefit	23 (2.4)		170 (12.78)		247 (16.25)		75 (9.7)		35 (3.29)		550 (9.75)				

^a^N/A: not applicable.

First, the group of patients who received a psychiatric diagnosis from the psychiatric department had the highest suicide survival rate (888/951, 93.4%). A total of 951 (16.86%) patients were included in this group, 63 (6.6%) of whom died by suicide. The median age was 32 (range21.0-47.0) years, and 432 (45.4%) patients were male. The number of people in their 10s and 20s was higher than that of people aged >60 years. A total of 322 (33.9%) patients were diagnosed with cancer, and the median CCI score was 0.0 (range 0.0-1.0). Among the 5 groups, they had the lowest rates of disability (56/951, 5.8%), medical benefit insurance (23/951, 2.4%), moderate/heavy smokers (441/951, 46.3%), and drinkers (442/951, 46.3%).

Second, the group of patients who received a psychiatric diagnosis from other departments and then from psychiatry had the highest rate of self-harm with fatal methods (877/1330, 65.94%). A total of 1330 (23.58%) patients were included in this group, 118 (8.87%) of whom died by suicide. The median age was 45.0 (range 30.0-59.0) years, and 504 (37.9%) patients were male. The proportion of people in their 40s (241/1330, 18.12%) was higher than in other age groups. Cancer and disability history were noted in 641 (48.20%) and 209 (15.73%) patients, respectively. The median CCI score was 1.0 (range 0.0-2.0). Regarding socioeconomic factors, 824 (61.96%) and 823 (61.88%) patients were moderate or heavy smokers and drinkers, respectively.

Third, the group of patients who received a psychiatric diagnosis from psychiatry and then from other departments included 1520 (26.95%) patients, 154 (10.13%) of whom died by suicide. The median patient age was 52 (range 38-68) years, and 562 (36.97%) patients were male. They reported a higher fatality rate (946/1520, 62.24%) in their DSH. The proportion of people in their 50s (311/1520, 20.46%) was higher than that of other age groups. They had the highest rates of cancer with 812 (53.42%) patients, disability with 291 (19.14%) patients, moderate smokers (772/1520, 50.79%), moderate drinkers (775/1520, 50.99%), and medical benefit insurance (247/1520, 16.25%) among the 5 groups.

Fourth, the group of patients who received a psychiatric diagnosis only from departments other than psychiatry included 775 (13.74%) patients, 100 (12.9%) of whom died by suicide. The median age was 53.0 (range 40.0-66.0) years, and 406 (52.4%) patients were male. The highest proportion of people in their 50s (22.7%) was among the 5 groups. A history of cancer and disability was noted in 350 (45.2%) and 118 (15.2%) patients, respectively. The median CCI score was 2.0 (range 0.0-3.0). Most of them had employee or local insurance (700/775, 90.3%), and they had the highest number of heavy drinkers (203/775, 26.2%) among the 5 groups.

Finally, the group of patients who did not have a psychiatric diagnosis from any department had the lowest suicide survival rate (81.5%). A total of 1064 (18.87%) patients were included in this group, 197 (18.52%) of whom died by suicide. The median age of the patients was 43.0 (range 29.0-54.0) years, and 669 (62.88%) patients were men. The rate of people in their 40s and 50s (413/1064, 38.82%) was higher than that in other age groups. A history of cancer and disability was noted in 363 (34.12%) and 73 (6.86%) patients, respectively. The median CCI score was 1.0 (range 0.0-2.0). Most of them had employee or local insurance (1029/1064, 96.71%) and had the highest number of heavy smokers (252/1064, 23.68%) among the 5 groups.

### Survival Rates of Individuals With DSH Behavior by Various Subgroups

**Figure 2:**
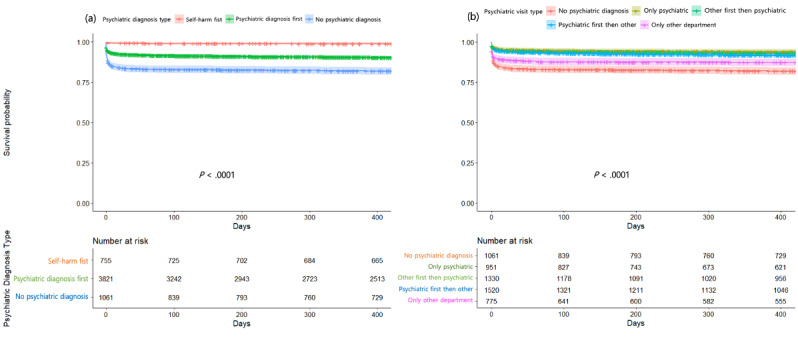
Kaplan-Meier curves displaying the estimated suicide survival probability for (a) 3 groups of patients whether they either did or did not receive a psychiatric diagnosis and whether they did receive a psychiatric diagnosis either before or after deliberated self-harm, as well as for (b) 5 groups of patients whether they did or did not receive a psychiatric diagnosis from psychiatric or other departments.

Temporal patterns were plotted using Kaplan-Meier survival curves, calculated separately for groups according to the time between self-harm and receiving a psychiatric diagnosis (3 groups) and for groups according to the departments providing psychiatric diagnosis (5 groups) in Figure 2. These survival curves indicated that there were significantly more incidences of death by suicide over time by days in the 1-year follow-up among the participants with *no psychiatric diagnosis* than in those with *self-harm first* (Figure 2A) and among the participants with *only other departments* than in those with *only psychiatric department* (Figure 2B).

### Risk Factors of Completed Suicide in Individuals With DSH Behavior

In the univariate Cox proportional hazards analysis, all variables showed a statistically significant impact on the suicide survival rate, except for the type of NHI (*P*=.39) and cancer history (*P*=.07; Table 3). Univariate Cox proportional hazards model indicated that higher severity of self-harm (HR 5.25, 95% CI 4.35-6.34; *P*<.001), male sex (HR 0.49, 95% CI 0.42-0.57; *P*<.001), older age (HR 1.04, 95% CI 1.03-1.04; *P*<.001), severe CCI (HR 1.51, 95% CI 1.43-1.59; *P*<.001), mild disability (HR 0.49, 95% CI 0.39-0.61; *P*<.001), severe smoking (HR 0.88, 95% CI 0.79-0.98; *P*=.02), severe drinking (HR 0.86, 95% CI 0.77-0.95; *P*=.004), no psychiatric diagnosis (HR 1.72, CI 1.45-2.04; *P*<.001), and no psychiatric visit (HR 0.66, 95% CI 1.47-2.25; *P*=.005) were significantly and independently associated with increased risk of suicide of intensive self-harm (Table 3).

**Table 3:** Cox proportional hazards regression of the association between self-harm-related potential risk factors and suicide-related mortality.

				Univariate analysis							Multivariate analysis				
				Coef^a^		HR^b^ (95% CI)		*P* value		Coef			HR (95% CI)		*P* value
Self-harm severity				1.659		5.25 (4.35-6.34)		<.001^c^		1.461			4.31 (3.55-5.26)		<.001^c^
**Demographic factors**															
	**Sex**														
		Female	N/A^d^		1 (reference)		N/A		N/A			1 (reference)		N/A	
		Male	0.716		0.49 (0.42-0.57)		<.001^c^		0.362			0.70 (0.59-0.83)		<.001^c^	
	Age			0.034		1.04 (1.03-1.04)		<.001^c^		0.042			1.04 (1.03-1.06)		<.001^c^
**Medical history–related factor**															
	Cancer			−0.149		0.86 (0.74-1.01)		.07		N/A			N/A		N/A
	Charlson comorbidity index			0.411		1.51 (1.43-1.59)		<.001^c^		0.087			0.92 (0.77-1.10)		.34
	**Disability status**														
		No	N/A		1 (reference)		N/A		N/A			1 (reference)		N/A	
		Mild	0.722		0.49 (0.39-0.61)		<.001^c^		0.160			0.85 (0.68-1.07)		.17	
		Severe	−1.024		0.36 (0.23-0.56)		<.001^c^		−0.563			0.57 (0.37-0.89)		.01^e^	
**Socioeconomic factor**															
	Smoking			0.129		0.88 (0.79-0.98)		.02^e^		0.272			0.76 (0.62-0.93)		.009^f^
	Drinking			0.156		0.86 (0.77-0.95)		.004^f^		0.288			0.75 (0.61-0.92)		.005^f^
	**National Health Insurance**														
		Medical benefit	N/A		1 (reference)		N/A		N/A			1 (reference)		N/A	
		Employee or local	−1.024		0.89 (0.67-1.17)		.39		N/A			N/A		N/A	
**Psychiatric diagnosis and psychiatry department–related factor**															
	**Psychiatric diagnosis type**														
		Psychiatric diagnosis first	N/A		1 (reference)		N/A		N/A			1 (reference)		N/A	
		Self-harm first	−1.729		0.18 (0.11-0.29)		<.001^c^		−1.865			0.15 (0.10-0.25)		<.001^c^	
		No psychiatric diagnosis	0.541		1.72 (1.45-2.04)		<.001^c^		0.608			1.84 (1.47-2.30)		<.001^c^	
	**Psychiatry visit type**														
		Psychiatry first, then other	N/A		1 (reference)		N/A		N/A			1 (reference)		N/A	
		Only psychiatry	−0.418		0.66 (0.49-0.88)		.005^f^		−0.380			1.46 (1.08-1.99)		.02^e^	
		Other first, then psychiatry	−0.143		0.87 (0.68-1.10)		.24		−0.104			1.11 (0.87-1.41)		.40	
		Only other departments	0.247		1.28 (1.00-1.65)		.05		0.262			1.30 (1.01-1.68)		.04^e^	
		No psychiatric diagnosis	N/A		1.82 (1.47-2.25)		<.001^c^		N/A			N/A		N/A	

^a^Coef: coefficient.

^b^HR: hazard ratio.

^c^*P*<.001.

^d^N/A: not applicable.

^e^*P*<.05.

^f^*P*<.01.

To jointly determine the impact on survival, 9 statistically significant variables were incorporated into the multivariate model. The factors that affected suicide are given in the following order based on the HR value: higher severity of self-harm (HR 4.31, 95% CI 3.55-5.26; *P*<.001), never visited a psychiatrist (HR 1.84, 95% CI 1.47-2.30; *P*<.001), psychiatric diagnosis from departments other than psychiatry (HR 1.30, 95% CI 1.01-1.68; *P*=.04), older age (HR 1.04, 95% CI 1.03-1.06; *P*<.001), severe smoking (HR 0.76, 95% CI 0.62-1.07; *P*=.009), severe drinking habits (HR 0.75; 95% CI 0.61-0.92; *P*=.005), male sex (HR 0.70, 95% CI 0.59-0.83; *P*<.001), and no severe disability (HR 0.57, 95% CI 0.37-0.89; *P*=.01). All these factors may have statistically significant effects on the high risk of suicide.

Psychiatric diagnosis and psychiatry department–related variables, as defined in our study, also had a statistically significant effect on the high risk of suicide. *No psychiatric diagnosis* indicates that *no clinical visit* had a statistically significant effect on low suicide survival (*P*<.001). *Only psychiatry visit* (HR 1.46, 95% CI 0.49-0.87; *P*=.02) and *only other departments* (HR 1.30, 95% CI: 1.01-1.68; *P*=.04) indicates that the proper treatment for self-harm had statistically significant effect on high suicide survival.

## Discussion

### Principal Findings

This study aimed to investigate the characteristics of individuals with DSH behavior using nationwide cohort data and to determine the factors that contribute to suicide-related deaths in this population. Such efforts could provide valuable information about individuals with DSH behavior who are especially susceptible to subsequent death by suicide in South Korea, the nation with the highest suicide rate among Organization for Economic Co-operation and Development countries [31]. We predicted that having a psychiatric diagnosis would act as a protective factor against suicide completion. This prediction was based on previous studies describing the relationship between mental health care access and suicide rates. On the basis of a recent study exploring national trends in mental health care among adults who previously attempted suicide, despite the increasing rate of suicide attempts, the use of services among those who attempted suicide did not increase; therefore, there is a great need to expand service accessibility [9]. Several studies have suggested that greater access to mental health services was associated with a reduced incidence of suicide. More access was measured in terms of living at a range of distances from the nearest mental health provider, the availability of specific services, or the density of mental health providers [44-46]. However, these studies did not differentiate between psychiatrists and nonpsychiatric physicians in their analyses. A study exploring short-term suicide risk after psychiatric hospital discharge showed a trend in which patients who had severe psychiatric conditions for which they were seen by psychiatrists and then admitted to the psychiatric ward committed suicide within a few months [47]. Despite conflicting previous findings, it is still unclear whether being seen by a mental health care professional reduces the rate of suicide-related death in individuals with DSH behavior.

The main finding of this study is that missing a psychiatric diagnosis and therefore missing an appointment with a psychiatrist may contribute to increased HRs independent of already known contributing factors such as male sex, old age, the use of fatal form of DSH, and physical disability. Unsurprisingly, the most important contributor to suicide was the use of a fatal method of self-harm, which has been repeatedly mentioned as a very important risk factor for suicide death in previous literature [3,5,6,11,14-19]. Having a psychiatric diagnosis, whether given by a psychiatrist or a nonpsychiatrist physician, was the second most important contributing factor for suicide-related deaths. In alignment with findings from other studies, having access to a hospital where the physician can evaluate one’s psychiatric conditions may reduce the risk of suicide in individuals who had or would engage in DSH [7]. More importantly, whether someone had an interview or a check-up with a psychiatrist may be more informative than any demographic or socioeconomic status information in predicting one’s chance of committing fatal forms of DSH, which could potentially lead to suicide death.

As there was a strong correlation between receiving a psychiatric diagnosis and suicide completion, it was deemed imperative to identify the characteristics of individuals who were at a very high risk of suicide death—individuals who had never received a psychiatric diagnosis. Among the 3 subgroups of DSH, the subgroup that had never encountered a medical professional for their mental health condition showed the highest percentage of deaths by suicide, and as expected, more than half of them used fatal methods of DSH (Table 1). From a socioeconomic standpoint, this subgroup consisted mostly of men in their 40s and 50s, who consumed heavy amounts of alcohol and received health insurance through Medicare (employment/regional health insurance). Some of these characteristics, such as male sex and substance abuse, are known risk factors for increased suicide rates [2]; some other characteristics, such as being middle-age and receiving Medicare, have not been associated with suicide deaths. The KNHIS provides mandatory public health insurance, offering coverage of health care services to almost 100% of South Koreans; 97% of South Koreans are covered by Medicare and 3% are covered by Medicaid [8]. Medicaid is provided to people whose income is insufficient to meet their needs and those of their families, and they are exempted from health insurance fees, whereas those with Medicare pay approximately 10% to 30% of their total medical expenses when using medical facilities. Hence, the type of health insurance is an indirect measure of socioeconomic status, where receiving Medicaid is associated with lower socioeconomic status. Surprisingly, compared with individuals who visited the hospital for psychiatric assessment, those who did not were mostly on Medicare. Our study’s finding is opposite to what previous studies have suggested: higher socioeconomic status, including higher levels of educational achievement, higher income, and employment, is associated with better health and lower risks of all-cause mortality, including deaths by suicide [14,48]. Medicare use may be a primary contributing factor of suicide death in these individuals with DSH behavior, as having to pay for additional medical expenses to be able to see a medical professional for mental health concern may have discouraged such pursuit. Higher socioeconomic status may also have contributed to not seeking psychiatric care; South Korea being notorious for its competitive work environment and demanding high performance and efficiency has shaped such an atmosphere where middle-age employees must hide weaknesses, such as having psychological difficulties.

Table 2 displays how seeing a psychiatrist as opposed to a nonpsychiatrist physician affected survival. For our subanalysis, we categorized individuals who had received a psychiatric diagnosis by the different hospital departments they paid visit to. Suicide-related death rates increased in a stepwise fashion. Notably, individuals who had at least 1 visit to the psychiatrist were mostly women, and this sex difference in psychiatric visits has been noted in previous literature [18]. In addition, individuals in their 20s were the largest age group for most visits to the psychiatric department. Although seeing a psychiatrist continues to be taboo for most older South Korean adults and older adults, the younger generations appear to be more willing to seek professional help and voice concerns about their mental health. This finding may also reflect the recent rise in the youth and young adult depression and anxiety cases in South Korea; this phenomenon has already been hinted in previous studies [32,49]. Another noteworthy finding is that individuals who had mild to severe physical disabilities belonged to subgroups that had received psychiatric diagnoses from psychiatry departments as well as other departments and showed a tendency to engage in more severe forms of DSH. Having a physical disability significantly increased one’s risk of DSH as well as death by suicide, in part by making them more susceptible to the deterioration of mental health [14,50]. It may be in the best interest of nonpsychiatric physicians to perform routine psychiatric evaluations and to actively consult with psychiatrists if their patients show signs of heightened anxiety and depression during treatment for their physical health conditions.

The total number of individuals with DSH behavior and suicide-related deaths was considerably small despite using NHI cohort data, which included all health-related information of the entire Korean population. According to the 2020 national statistics, 25.7 per 100,000 persons died of suicide attempts [51]; hence, the data set we used in our analyses did not account for all DSH and deaths by suicide cases in South Korea. We infer that this shortage of data could be accounted for by 2 main reasons: (1) suicide and self-harming behavior are major taboos in South Korean society and (2) suicide or self-harm–related diagnosis would disqualify one from reaping medical insurance benefits. It is suspected that most bereaved families as well as the attempters who survived for the reasons mentioned above would strongly refuse to receive a diagnosis for DSH. This would then put pressure on physicians to forgo, including self-harm–related ICD-10 diagnosis codes in the official hospital records. Thus, although high suicide rates in South Korea are readily recognized by the media and the Korean Statistical Information Service, it is not possible to acquire patient information from hospital electronic medical records and the NHI database for every DSH case. Nevertheless, the data set used for our analyses was technically gathered from every hospital in South Korea; therefore, it should represent the DSH population in South Korea to a reasonable extent. To that end, although our study results should be interpreted with careful consideration, our analyses have important clinical value.

### Conclusions

In conclusion, we investigated and described the characteristics of individuals with DSH behavior using South Korea’s nationwide cohort data and found that receiving proper assessment for psychiatric conditions, especially from a psychiatrist, reduced suicide deaths. In this modern age, we believe that the pattern we observed in individuals with DSH behavior whose deaths were suicide related is not a phenomenon solely pertinent to South Korea but also applicable in other parts of the world. Physicians, irrespective of their culture and language, may take this into consideration when screening for individuals at high risk of DSH and suicide. We also found some important clinical features of individuals with physical disabilities associated with suicidality, requiring mental health condition or suicide risk assessment in nonpsychiatric clinical settings; such information could be used to develop guidelines to assist nonpsychiatric health care professionals. Future studies using larger cohort data are necessary to further explore and replicate the findings of this study.

## Appendix

Multimedia Appendix 1 Variable selection based on literature review.

## Abbreviations

CCI: Charlson comorbidity index
DSH: deliberate self-harm
HR: hazard ratio
ICD-10: International Classification of Diseases, 10th Revision
KNHIS: Korean National Health Insurance Service
NHI: National Health Insurance
