# Supplementary material for: Investigation of the Relationship Between Psychiatry Visit and Suicide After Deliberate Self-harm: Longitudinal National Cohort Study
Source: JMIR Public Health Surveill. 2023 Apr 12;9:e41261. doi: 10.2196/41261 (PMC10134021; doi:10.2196/41261)
Supplement: Multimedia Appendix 1 [file publichealth_v9i1e41261_app1.docx]

Multimedia Appendix 1. Variable selection based on literature review

| *Variables* | | *Skegg K.^[[1]](#footnote-1)^* | *Olfson et al.^[[2]](#footnote-2)^* | *Crump et al.^[[3]](#footnote-3)^* | *Our study* | |
| --- | --- | --- | --- | --- | --- | --- |
| Demographic profile | age | O | O | O | O | age |
|  | sex | O | O | O | O | sex |
|  | race/ethnicity |  | O | O | X |  |
|  | income | O |  | O | Δ | Insurance type |
|  | location (urban/rural) |  |  | O | X |  |
|  | employment status |  |  | O | X |  |
|  | education level |  |  | O | X |  |
|  | marital status |  |  | O | X |  |
| Social and family environment | adverse childhood experience | O |  |  | X |  |
|  | social support | O |  |  | X |  |
|  | religious affiliation | O |  |  | X |  |
|  | cultural norms | O |  |  | X |  |
| Psychiatric disorders | depression | O | O | O | Δ | psychiatric diagnosis |
|  | bipolar | O | O | O | Δ | psychiatric diagnosis |
|  | anxiety | O | O | O | Δ | psychiatric diagnosis |
|  | schizophrenia | O | O | O | Δ | psychiatric diagnosis |
|  | alcohol use | O | O | O | O | drinking |
|  | drug use | O | O | O | O | smoking |
|  | personality disorder | O | O | O | Δ | psychiatric diagnosis |
|  | other mental disorder | O | O | O | Δ | psychiatric diagnosis |
| Self-harm treatment setting | outpatient |  | O |  | Δ | psychiatric visit |
|  | inpatient |  | O |  | Δ | psychiatric visit |
|  | emergency |  | O |  | Δ | psychiatric visit |
|  | self-harm method |  | O |  | Δ | DSH fatality |
| Somatic disorders | asthma |  |  | O | Δ | CCI |
|  | cancer |  |  | O | O | cancer |
|  | diabetes mellitus |  |  | O | Δ | CCI |
|  | ischemic heart disease |  |  | O | Δ | CCI |
|  | stroke |  |  | O | Δ | CCI |
|  | COPD |  |  | O | Δ | CCI |
| Psychological characteristics | Impulsiveity | O |  |  | X |  |
|  | poor problem-solving | O |  |  | X |  |
| Neurobiological and genetic aspects | Inherited vulnerability of serotonin system | O |  |  | X |  |
| Situational factors | adverse life events | O |  |  | X |  |
|  | media influence | O |  |  | X |  |
|  | awareness of self-harm of others | O |  |  | X |  |
|  | intoxication | O |  |  | X |  |
| Physical illness | disability | O |  |  | O | Disability |

O: exact matching; Δ: substitute matched; X: not matched

Except for one (e.g., race/ethnicity) not provided in KHNIS, a total of 11 variables were selected, consisting of six matching variables including age, sex, drinking, smoking, cancer, and disability, and five variables (e.g., income/insurance type, psychiatric disorders/psychiatric diagnosis, self-harm treatment setting/psychiatric visit, and self-harm method/DSH, and somatic disorders/CCI) that were not clearly matched but could be substituted with the same semantically.

1. Skegg, Keren. "Self-harm." *The Lancet* 366.9495 (2005): 1471-1483. [↑](#footnote-ref-1)
2. Olfson, Mark, et al. "Suicide following deliberate self-harm." *American Journal of Psychiatry* 174.8 (2017): 765-774. [↑](#footnote-ref-2)
3. Crump, Casey, et al. "Sociodemographic, psychiatric and somatic risk factors for suicide: a Swedish national cohort study." *Psychological medicine* 44.2 (2014): 279-289. [↑](#footnote-ref-3)
